# Supplementary figures and images for: Human Papillomavirus Type 16 Stimulates WAVE1- and WAVE2-Dependent Actin Protrusions for Endocytic Entry
Source: Viruses. 2025 Apr 8;17(4):542. doi: 10.3390/v17040542 (PMC12031361; doi:10.3390/v17040542)

**Figure S1**

A)

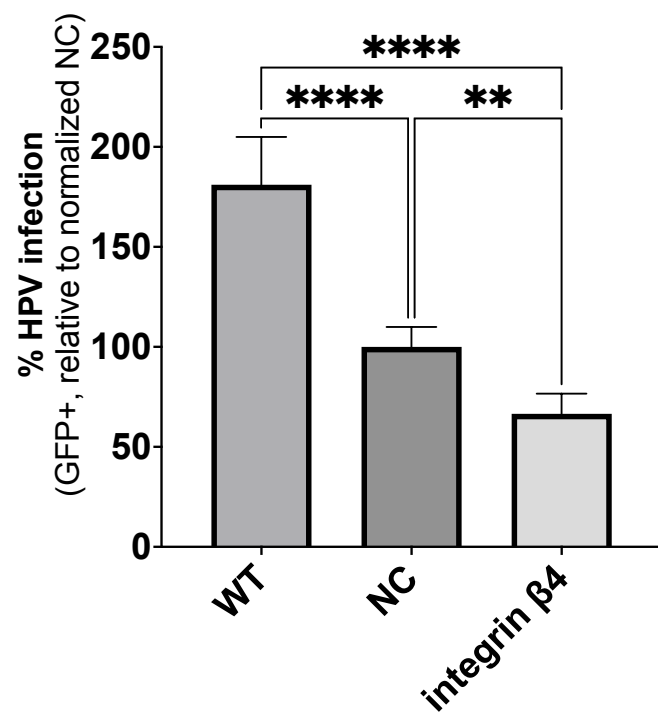

B)

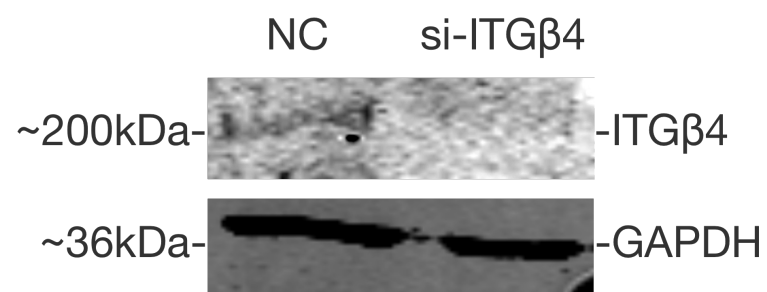

**Figure S2**

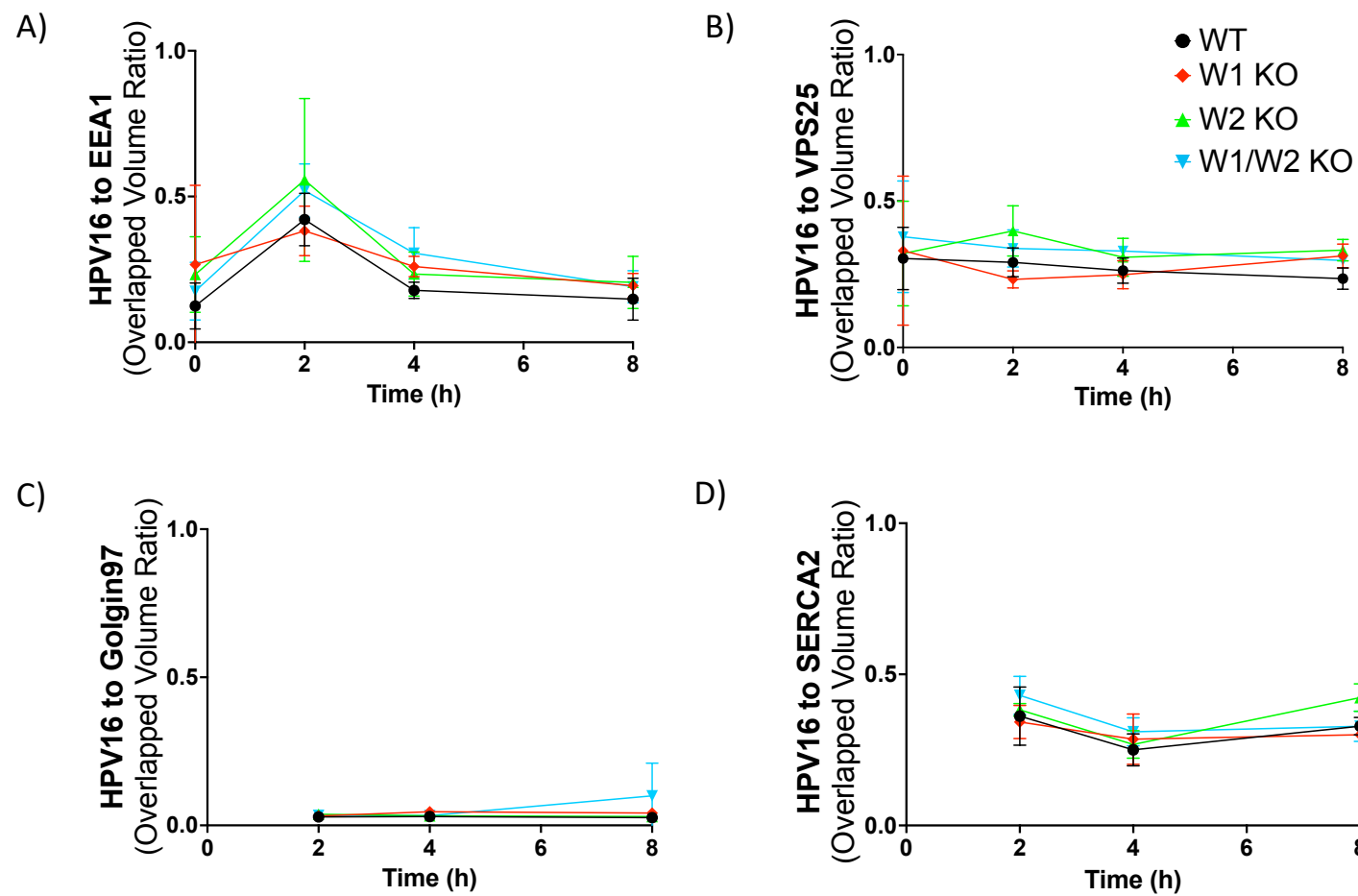

**Figure S3**

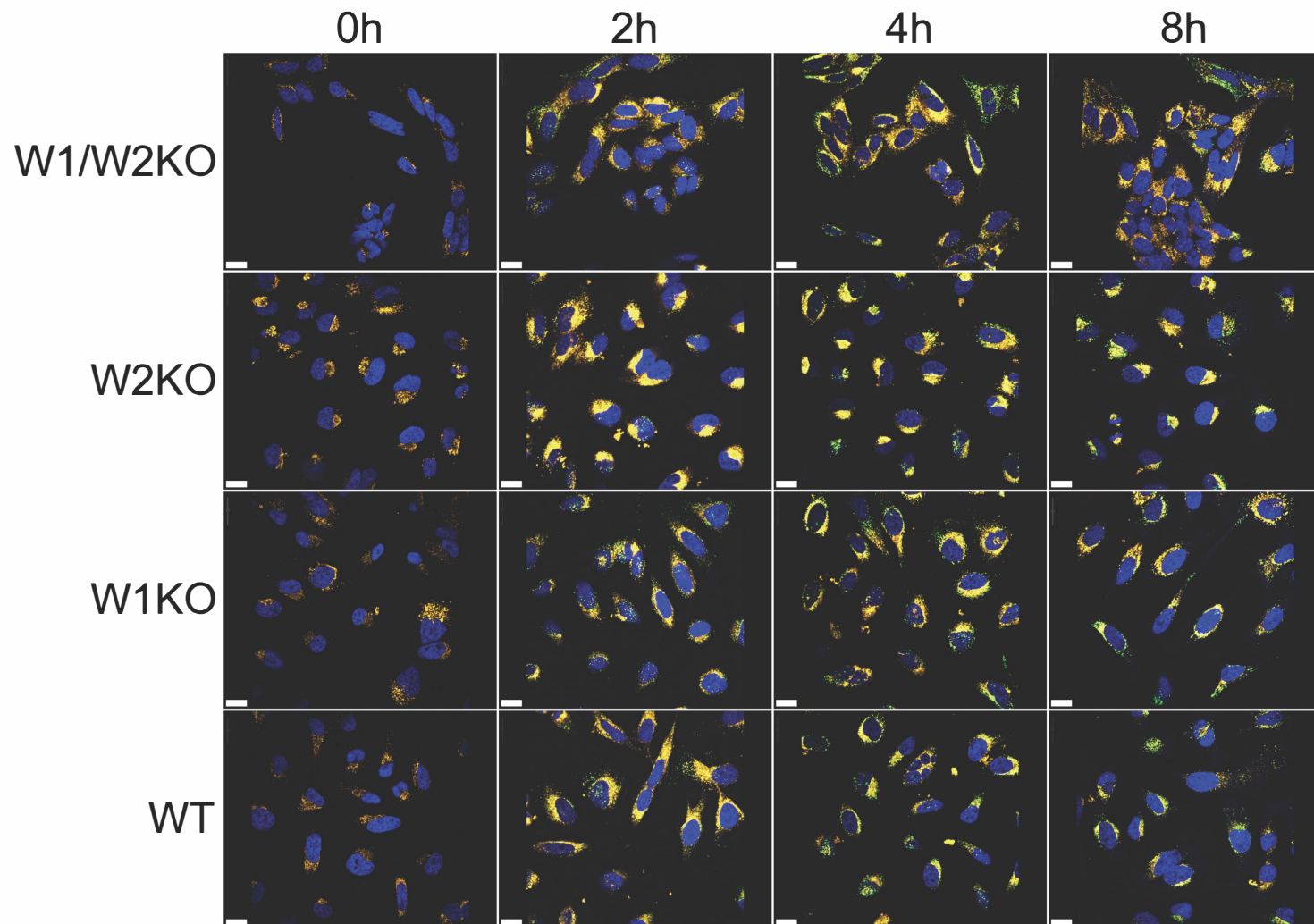

Figure S4

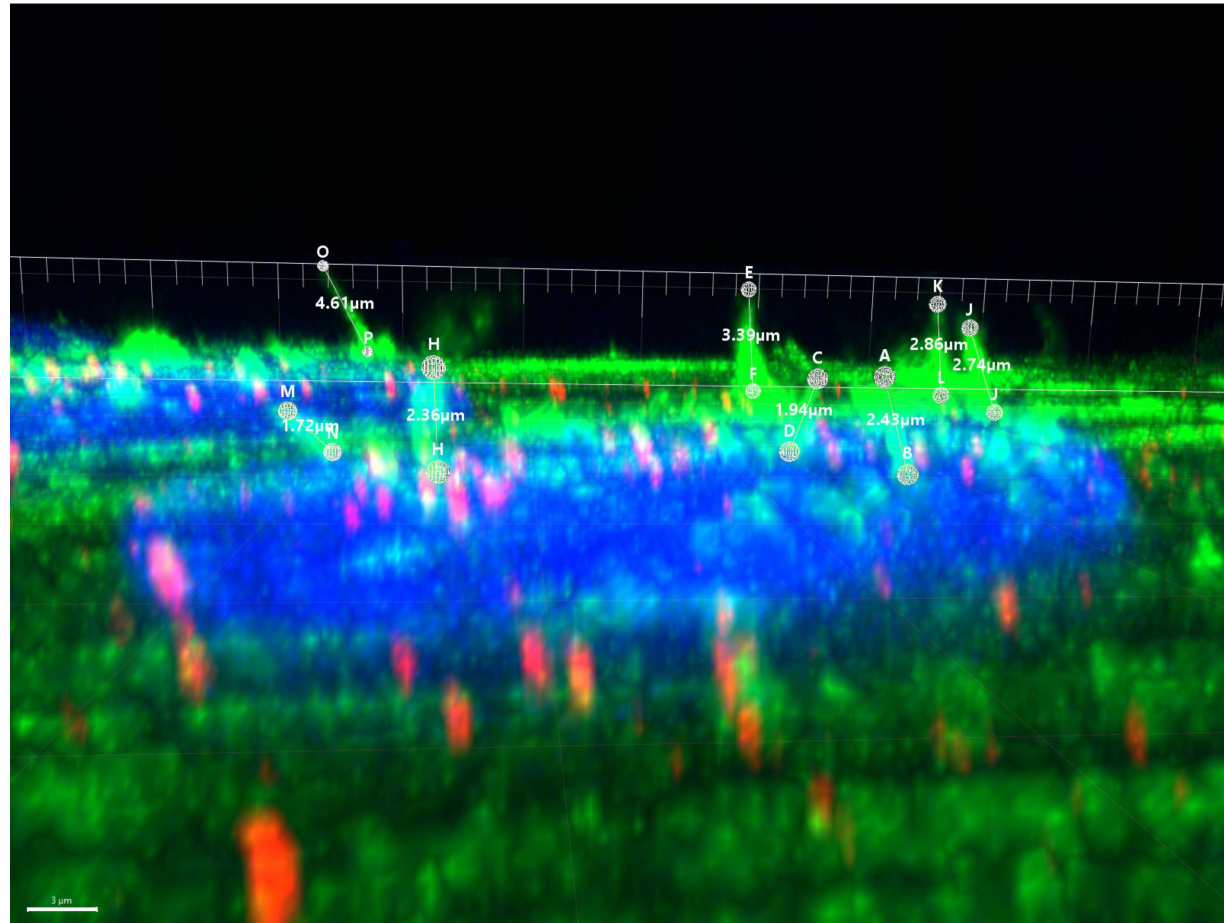

Supplement: Supplementary file 1 [file viruses-17-00542-s001.zip › viruses-3524801-supplementary.pdf]
